# Inhibition of MAT2A Impairs Skeletal Muscle Repair Function

**DOI:** 10.3390/biom14091098

**Published:** 2024-09-02

**Authors:** Wanli Xiao, Tian-E Huang, Jing Zhou, Benhui Wang, Xiang Wang, Weirong Zeng, Qiquan Wang, Xinqiang Lan, Yang Xiang

**Affiliations:** Metabolic Control and Aging—Jiangxi Key Laboratory of Aging and Diseases, Human Aging Research Institute (HARI), School of Life Science, Nanchang University, Nanchang 330031, China; 352428819003@email.ncu.edu.cn (W.X.); hte0407@email.ncu.edu.cn (T.-E.H.); jingzhou2303@163.com (J.Z.); wbhxm8@163.com (B.W.); xiangwang@email.ncu.edu.cn (X.W.); 18370384928@163.com (W.Z.); wangqiquan@ncu.edu.cn (Q.W.); lanxinqiang@ncu.edu.cn (X.L.)

**Keywords:** *Mat2a*, C2C12, skeletal muscle, Fas, regeneration

## Abstract

The regenerative capacity of muscle, which primarily relies on anabolic processes, diminishes with age, thereby reducing the effectiveness of therapeutic interventions aimed at treating age-related muscle atrophy. In this study, we observed a decline in the expression of methionine adenosine transferase 2A (MAT2A), which synthesizes S-adenosylmethionine (SAM), in the muscle tissues of both aged humans and mice. Considering MAT2A’s critical role in anabolism, we hypothesized that its reduced expression contributes to the impaired regenerative capacity of aging skeletal muscle. Mimicking this age-related reduction in the MAT2A level, either by reducing gene expression or inhibiting enzymatic activity, led to inhibiting their differentiation into myotubes. *In vivo*, inhibiting MAT2A activity aggravated BaCl_2_-induced skeletal muscle damage and decreased the number of satellite cells, whereas supplementation with SAM improved these effects. RNA-sequencing analysis further revealed that the Fas cell surface death receptor (Fas) gene was upregulated in *Mat2a*-knockdown C2C12 cells. Suppressing MAT2A expression or activity elevated Fas protein levels and increased the proportion of apoptotic cells. Additionally, inhibition of MAT2A expression or activity increased p53 expression. In conclusion, our findings demonstrated that impaired MAT2A expression or activity compromised the regeneration and repair capabilities of skeletal muscle, partially through p53–Fas-mediated apoptosis.

## 1. Introduction

Skeletal muscle regeneration is a crucial biological process for repairing skeletal muscle injuries. However, aging makes skeletal muscle more susceptible to injuries and diminishes its capacity to repair itself [1,2]. The repair and regenerative activity of skeletal muscle is closely linked to its metabolism [3,4]. Additionally, anabolic metabolism plays a crucial role in the repair of skeletal muscle; however, aging leads to anabolic resistance, which is defined as a diminished response of the muscle to anabolic stimuli, such as protein intake and physical activity, often leading to reduced muscle growth and repair capacity [5]. Despite ongoing efforts to develop interventions, such as nutritional supplementation, resistance training, aerobic exercise, and natural small-molecule interventions [6], the decline in skeletal muscle regeneration and repair capacity remains insufficiently addressed, particularly concerning the proliferation and differentiation of satellite cells and myoblasts. Skeletal muscle repair and regeneration depend on the proliferation and differentiation of satellite cells and myoblasts. Derived from skeletal muscle satellite cells, myoblasts can differentiate into muscle-like cells and display myogenic characteristics. However, the quantity and functionality of myoblasts gradually decline, impairing the repair process after muscular damage [7]. In aging skeletal muscle, satellite cells display defective and compromised regenerative capacities, rendering them ineffective at sustaining muscle regeneration and functionality [8]. Although aging is recognized as a risk factor for impaired skeletal muscle regeneration and repair, the precise underlying mechanisms remain elusive. Interventions, such as mitochondrial transplantation, reduction of cellular inflammatory responses, antioxidants, and autophagy inducers, have been shown to promote skeletal muscle regeneration [9,10,11,12]. Overall, most interventions actually achieve their effects through the modulation of skeletal muscle metabolism.

Metabolic changes in aged skeletal muscle are associated with decreased muscle mass and function. Impaired glucose metabolism, fatty acid metabolism, and redox homeostasis decrease the bioavailability of long-chain acylcarnitine, amino acids, and free fatty acids [13,14]. Human muscle biopsy data indicate that baseline levels of tricarboxylic acid cycle metabolites, including succinic acid, fumaric acid, and 2-ketoglutaric acid, are reduced in the elderly. Additionally, lower levels of adenosine triphosphate (ATP), adenosine diphosphate (ADP), branched-chain amino acids, and acylcarnitine in aged muscle suggest impaired mitochondrial function or a reduced number of mitochondria [15]. Studies have shown that these metabolic changes can be improved by exercise. Recent studies have found that leucine increases muscle mass, and arginine and lysine metabolites decrease in aging muscle [14]. Supplementation with essential amino acids has been found to enhance muscle mass, strength, and function in the elderly [16,17,18]. Among these metabolic changes and interventions, alterations in anabolic metabolism play the most crucial role in the repair and regeneration of skeletal muscle.

S-adenosylmethionine (SAM), which is essential in anabolic processes, decreases in skeletal muscle with aging [14,19,20]. As the primary methyl donor in mammalian cells and an aminopropyl donor for polyamines, SAM’s reduction underscores the broader impact of anabolic deficits on muscle aging [21]. This is further complicated by the role of methionine adenosine transferase 2A (MAT2A), a rate-limiting enzyme in the methionine cycle responsible for catalyzing SAM synthesis from methionine and ATP [22]. The enzyme methionine adenosine transferase (MAT) possesses two subtypes in mammals, namely, MAT1A and MAT2A. Specifically, MAT1A is primarily expressed in the liver, while MAT2A is expressed in extrahepatic tissues and non-parenchymal cells of the liver [23,24]. MAT2A knockdown can effectively suppress the proliferation and induce the apoptosis of hepatoma cells [25]. Moreover, inhibition of MAT2A has been observed to induce cell cycle arrest and DNA damage in colorectal cells [26]. Nevertheless, the role and mechanism of MAT2A in skeletal muscle repair have not been reported to date.

With advancing age, the population of skeletal muscle satellite cells undergoes a gradual decline or impairment due to apoptosis and senescence [7]. Fas cell surface death receptor (Fas) is a death receptor that can bind to the Fas ligand (FasL), thereby activating caspase-8 and inducing apoptosis [27,28]. Moreover, the transcription level of Fas is regulated by p53 [29,30]. The p53 plays a crucial role in the regulation of cellular proliferation and apoptosis [30].

In this study, we performed a collaborative analysis of transcriptome-sequencing data from muscle tissues of mice at different ages and humans of varying ages. Interestingly, we found that the mRNA levels of *MAT2A* were reduced in aged muscle tissues from both mice and humans. Considering the altered expression of MAT2A and its pivotal role in anabolic processes, we hypothesized that the reduction in MAT2A expression contributes to the impaired regenerative capacity of aging skeletal muscle. Further experiments showed that knocking down MAT2A enhanced Fas expression, led to apoptosis in myoblasts, and inhibited their differentiation. Since myoblast differentiation is crucial for skeletal muscle repair, we investigated the effect of MAT2A on skeletal muscle repair following injury. Our findings demonstrated that MAT2A accelerated skeletal muscle repair after injury and expedited the recovery of muscle function.

## 2. Material and Methods

### 2.1. Cell Culture and Treatment

Mouse myoblast (C2C12) cells (Procell, Wuhan, Hubei, China) were cultured in DMEM (Thermo Fisher Scientific, Waltham, MA, USA), supplemented with 10% fetal bovine serum (HyClone, Logan, UT, USA). The cells were maintained at a temperature of 37 °C in a 5% CO_2_ environment. When the cells reached a confluence of 70%~80%, they were washed twice with PBS. Subsequently, the medium supplemented with 2% horse serum (Gibco, Grand Island, NY, USA) was replaced with DMEM to facilitate myotube differentiation. The cellular states at day 1, day 3, day 5, and day 7, respectively, were examined and documented. The compound PF9366, as a potent inhibitor of MAT2A, effectively suppressed its enzymatic activity [31]. When the cell confluency reached around ~80%, the cells were treated with 10 μM of PF9366 (Sigma-Aldrich, St. Louis, MO, USA) for 48 h and used for further experiments.

### 2.2. Mouse Models 

C57BL/6 mice obtained from the Model Animal Research Center of Nanchang University were housed under specific pathogen-free conditions with ad libitum access to sterile rodent chow and drinking water. Male mice at the age of 4 months (young) and 26 months (elderly) were used for RNA sequencing or constructing skeletal muscle injury models. 

The drug was administered via intramuscular injection. A volume of 50 μL of 1.2% BaCl_2_ was injected into the tibialis anterior (TA) of mice using multiple injections. Saline was used as the control. The mice (4 months) were treated with the drug and sacrificed after 7 days to collect samples. After a 48 h treatment with BaCl_2_, the mice were administered an intramuscular injection of 2 μM of a PF9366 or SAM (Sigma-Aldrich, St. Louis, MO, USA) supplement through drinking water.

### 2.3. siRNA Transfection

The cells were seeded in a six-well plate and cultured until reaching a confluence of 70%~80%. Knockdown of target genes was achieved by transfecting cells with 50 nM of small interfering RNA (siRNA) utilizing Lipofectamine 2000 (Thermo Fisher Scientific, Waltham, MA, USA) for 48 h. The siRNAs were synthesized by GenePharma (Suzhou, Jiangsu, China), with their sequences presented as follows: si*Mat2a*-1, 5′-GUC CAC ACG AUU GUU AUA UTT-3′; si*Mat2a*-2, 5′-GGG AUC UGG AUC UGA AGA ATT-3′; NC, 5′-UUC UCC GAA CGU GUC ACG UTT-3′.

### 2.4. Total RNA Extraction

Cultured cells or mouse tissues were exposed to Trizol reagent treatment (BMASSAY®, Beijing, China), followed by centrifugation at 13,000× *g* for 5 min at 4 °C. Subsequently, the resulting supernatant was mixed with chloroform and underwent another round of centrifugation to isolate RNA in the aqueous phase through isopropanol treatment at −20 °C for 20 min. Washing and resuspension procedures were performed on the obtained RNA pellet using similar centrifugation conditions to those mentioned earlier. The total RNAs were dissolved in DEPC-treated water and the RNA concentration was determined using a NanoDrop (Thermo Fisher Scientific, Waltham, MA, USA).

### 2.5. Real-Time Quantitative PCR (qPCR) 

Total RNA was reverse transcribed into complementary DNA (cDNA) by the reverse transcriptase kit (Zomanbio, Beijing, China). qPCR was performed using M5 HiPer Realtime PCR Super mix (Mei5bio, Beijing, China) on the qTOWER3 G Real-Time PCR System (Analytik Jena, Jena, Germany). The following sequences of primers were utilized: *Mat2a*, forward-5′-ATC AGC AAA CAG GAA TAC G-3′ and reverse-5′-TGA CCT TCC CCT ACA GAC T-3′; *p21*, forward-5′-CCA TTC CTT TGA TGA CGC-3′ and reverse-5′-ATG CTG ACG GTG AAC TCG-3′; *p53*, forward-5′-TTG AAG GCC CAA GTG AAG-3′ and reverse-5′-TGA GAA GGG ACA AAA GAT GA-3′; *Fas*, forward-5′-CAC CCT GAC CCA GAA TAC C-3′ and reverse-5′-TCT CCT TTT CCA GCA CTT T-3′; *Tspan32*, forward-5′-GTA TTG CTT CCA TCC TGA CC-3′ and reverse-5’-GAG TAT CTG CCT TTT CTA TCC A-3′; *Actin*, forward-5′-GGG AGT AAT GGT TGG AAT G-3′ and reverse-5′-AAG CTC GTT ATA GAA AGA GTG G-3′. The Ct values were obtained by analyzing the amplification curve, and the gene expression levels were normalized to *Actin* levels using the 2^−ΔΔCt^ method.

### 2.6. RNA-Sequencing Analysis

The sequencing of the transcriptome in mouse muscle tissue was conducted by BGI (Shenzhen, Guangdong, China). The old and young males’ muscle transcriptome data (GSE159217) were from the publicly accessible Gene Expression Omnibus (GEO) (www.ncbi.nlm.nih.gov/geo/query/acc.cgi, accessed on 20 October 2021). Homologous genes were annotated based on sequences found in the Ensembl database (http://asia.ensembl.org/, accessed on 20 October 2021). The GRCh38.p14 annotation was employed for the characterization of genes in the human transcriptome. The GRCm39 annotation was employed for the characterization of genes in the mouse transcriptome. DESeq2 (v1.4.5) was employed for differential expression analysis, with a threshold of q_value < 0.05, to determine differential expression. The gene expression data were used to construct a heatmap using pheatmap (v1.0.12). The biological processes of differentially expressed genes (DEGs) were annotated using Gene Ontology (GO) enrichment analysis in the Gene Ontology resource (www.geneontology.org, accessed on 28 October 2021).

### 2.7. Western Blot Analysis

Protein extraction was performed using RIPA buffer (Solarbio, Beijing, China). In summary, after lysing the cells or tissues with RIPA for 1 h, they were centrifuged at 13,000× *g* for 5 min at 4 °C, and then the supernatant was collected. The protein concentration was determined by the BCA protein assay kit (Beyotime, Shanghai, China). Proteins were separated by SDS-PAGE and subsequently transferred onto PVDF membranes (Millipore, Billerica, MA, USA). The primary and secondary antibodies used in the assay were as follows: MAT2A (1:1000, Proteintech, Wuhan, Hubei, China), Fas (1:1000, Proteintech, Wuhan, Hubei, China), p53 (1:1000, CST, Danvers, MA, USA), cleaved caspase-3 (1:1000, CST, Danvers, MA, USA), GAPDH (1:10,000, Proteintech, Wuhan, Hubei, China), HRP goat anti-rabbit IgG (1:10,000, Proteintech, Wuhan, Hubei, China), and HRP goat anti-mouse IgG (1:10,000, Proteintech, Wuhan, Hubei, China). The protein bands were examined by the SuperSignal ECL detection kit (Beyotime, Shanghai, China) on the Tanon 5200 Chemiluminscent and Fluorescent Imaging System (Tanon, Shanghai, China).

### 2.8. Histochemistry and Immunofluorescence

H&E staining was used to analyze the structure of skeletal muscle to assess injury. Immunofluorescence was utilized to assess the protein expression in cells or tissues. In summary, the fixation of cells or tissue sections were fixed with 4% (vol/vol) paraformaldehyde for 10 min at room temperature. Subsequently, permeabilization was performed with 0.25% Triton X-100 for 10 min at room temperature. The samples were blocked and then incubated with a primary antibody, including dystrophin (1:100, Proteintech, Wuhan, Hubei, China), PAX7 (1:50, Thermo Fisher Scientific, Waltham, MA, USA), and Ki67 (1:100, Abcam, Cambridge, UK). The secondary antibody was Cy3-labeled donkey anti-rabbit IgG (1:200, Biolegend. San Diego, CA, USA). Hochest3342 (BMASSAY®, Beijing, China) was used to stain nuclei. The sections were observed under a confocal microscope (LSM800, Carl Zeiss, Oberkochen, Germany).

### 2.9. Measurement of SAM Production

The levels of SAM in the lysate of cells or skeletal muscle were measured using an ELISA kit (BMASSAY®, Beijing, China). Following the recommended procedures provided by the manufacturer, 50 μL of C2C12 cell lysate was added to a SAM conjugate-coated plate and incubated at room temperature for 10 min. Then, 50 μL of anti-SAM antibody was added and incubated at room temperature for 1 h. The color-rendering operation was performed in accordance with the manufacturer’s instructions. Subsequently, the levels of SAM were determined by measuring the optical density at a wavelength of 450 nm using the SpectraMax i3x (Molecular Devices, San Jose, CA, USA).

### 2.10. Apoptosis Analysis 

The quantification of cellular apoptotic activity was performed using an Annexin V-FITC apoptosis detection kit (Beyotime, Shanghai, China) according to the manufacturer’s instructions. Initially, the cells were washed with pre-cooled PBS and then suspended in a solution of Annexin V binding buffer. Subsequently, the cell suspension was incubated with Annexin V-FITC on ice for 15 min under dark conditions, followed by a 5 min incubation with PI. Finally, flow cytometry analysis (BD Bioscience in Franklin Lakes, NJ, USA) was performed to evaluate the treated cells. The proportion of apoptotic cells was analyzed by the FACSverse instrument (BD Biosciences, San Jose, CA, USA).

### 2.11. MTT Assay

C2C12 cells (treated with si*Mat2a* or PF9366) were seeded in a 96-well plate at a concentration of 5 × 10^3^ cells/well. After the cells were treated for 48 h, 10 μL of the MTT labeling reagent (Beyotime, Shanghai, China) was added to each well. The 96-well plate was incubated for 4 h at a temperature of 37 °C in a 5% CO_2_ environment. Then, 100 μL of the solubilization solution was added into each well. The 96-well plate was incubated at 37 °C for another 4 h. The absorbance of the 96-well plate was measured at a wavelength of 560 nm using the SpectraMax i3x (Molecular Devices, San Jose, CA, USA).

### 2.12. Statistical Analysis

The statistical analysis was performed using GraphPad Prism 10.0 software (La Jolla, CA, USA). The means ± SD of all values are represented in the results. A one-way ANOVA with Dunnett’s multiple comparison test was conducted to compare three or more groups, and a two-tailed Student’s *t*-test was used for comparing between two groups. For multiple comparisons involving two variables, a two-way ANOVA was utilized. A statistical significance level of *p* < 0.05 indicated significance.

## 3. Results

### 3.1. MAT2A Is Downregulated in the Muscle Tissue of Elderly Humans and Mice

**Figure 1:**
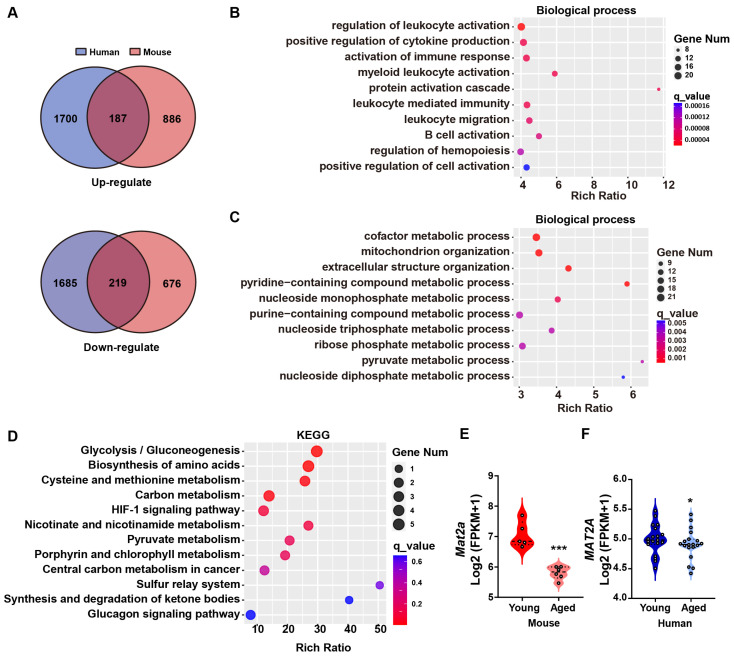
Decreased *MAT2A* expression in the aged skeletal muscle of humans and mice. (**A**) Venn diagram of age-related DEGs in the skeletal muscle of humans and mice, *p*-adjust < 0.05. (**B**) GO enrichment of upregulated genes in the skeletal muscle, q_value < 0.05. (**C**) GO enrichment of downregulated genes in the skeletal muscle, q_value < 0.05. (**D**) KEGG enrichment of cofactor metabolism-related genes. (**E**) *Mat2a* expression in the skeletal muscle of young mice (3 months, n = 5) and elderly mice (26 months, n = 5). (**F**) *MAT2A* expression in the skeletal muscle of young humans (19~25 years, n = 20) and elderly humans (66~71 years, n = 18). Statistical analysis was performed using the Student’s *t*-test. Data are presented as mean ± SD. * *p* < 0.05, *** *p* < 0.001.

Muscle mass and function rely on regeneration. However, the decline in muscle regeneration caused by aging leads to reduced muscle mass and decreased muscle strength [32]. In relation to skeletal muscle regeneration, the expression of Pax7, which is responsible for the proliferation of muscle satellite cells, and Myogenic Factor 5 (Myf5) and Myogenic Differentiation 1 (MyoD), which are responsible for their differentiation, all decrease in elderly skeletal muscle [33,34]. However, the role of metabolism-related genes in muscle regeneration remains unclear. Therefore, we performed transcriptome sequencing of quadriceps muscles of mice and conducted a comparative analysis with publicly available young/elderly humans’ vastus lateralis transcriptome data (GSE159217), with a particular focus on metabolic pathways. The vastus lateralis is one of the components of the quadriceps [35]. Homologous genes were annotated based on sequences retrieved from the Ensembl database, revealing that 71.91% of mouse genes exhibited presence in the human transcriptome dataset (Supplementary Table S1). We comprehensively analyzed the transcriptome data from the vastus lateralis of young/elderly humans and quadriceps muscles of mice and identified 187 upregulated genes and 219 downregulated genes (*p*-adjust < 0.05; Figure 1A; gene descriptions and data are shown in Supplementary Tables S2 and S3). Gene Ontology (GO) enrichment analysis showed that the upregulated genes were enriched in immunity- and inflammation-related processes, consistent with previously reported increases in inflammation in elderly muscle in humans (Figure 1B) [36]. Meanwhile, downregulated genes were related to metabolic processes, with ‘cofactor metabolic processes’ ranking first (Figure 1C). Cofactors play a crucial role in energy metabolism and facilitate redox reactions needed for the biosynthesis of biomolecules [37]. Therefore, we performed Kyoto Encyclopedia of Genes and Genomes (KEGG) enrichment on DEGs enriched in cofactor metabolism and found that *MAT2A* is involved in amino acid metabolism, particularly in cysteine and methionine metabolism (Figure 1D). Among the genes in this pathway, *Mat2a* expression exhibited the greatest reduction in skeletal muscle of old mice (Figure 1E). The decreased expression of *MAT2A*, which can be replicated in elderly humans, as shown in Figure 1F, indicated a conserved cross-species decline in skeletal muscle, suggesting it likely plays a significant biological role.

### 3.2. Knockdown of Mat2a Inhibited the Differentiation of Myoblasts

**Figure 2:**
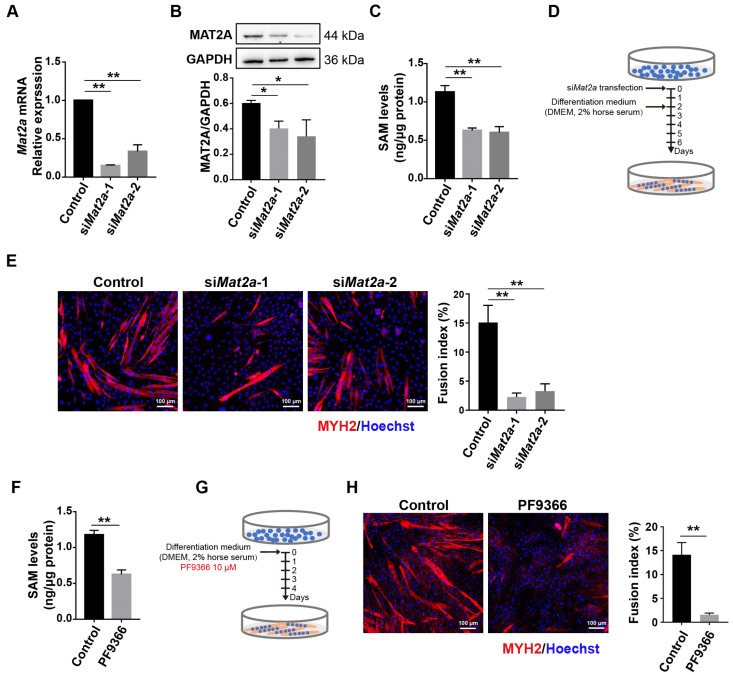
Inhibition of MAT2A expression or activity hindered differentiation of C2C12 cells. (**A**) qPCR for the mRNA expressions of *Mat2a* in C2C12 cells treated with si*Mat2a* for 48 h. Statistical analysis by one-way ANOVA. (**B**) Western blotting for the protein expression of MAT2A in C2C12 cells treated with si*Mat2a* for 48 h. Protein levels, normalized to GAPDH, were estimated by densitometry. Statistical analysis by one-way ANOVA. (**C**) ELISA for SAM levels in C2C12 cells treated with si*Mat2a* for 48 h. Statistical analysis by one-way ANOVA. (**D**) The C2C12 cells were transfected with si*Mat2a* for 48 h and then differentiated in DMEM supplemented with 2% horse serum for 4 days. (**E**) Immunofluorescence staining for MYH2 (red) and nuclei (blue) after 4 days of induced differentiation in C2C12 cells treated with si*Mat2a*. Scale bar: 100 µm. Quantification of the fusion index after these treatments is shown in the histogram. Statistical analysis by one-way ANOVA. (**F**) ELISA for SAM levels in C2C12 cells treated with PF9366 for 48 h. Statistical analysis was conducted using the Student’s *t*-test. (**G**) The C2C12 cells were treated with PF9366 and then differentiated in DMEM supplemented with 2% horse serum for 4 days. (**H**) Immunofluorescence staining for MYH2 (red) and nuclei (blue) after 4 days of induced differentiation in C2C12 cells treated with PF9366. Scale bar: 100 µm. Quantification of the fusion index after these treatments is shown in the histogram. Statistical analysis was conducted using the Student’s *t*-test. Each assay was conducted in triplicate. Data are presented as mean ± SD. * *p* < 0.05, ** *p* < 0.01 vs. control.

Myoblasts play a crucial role in the regeneration of skeletal muscle in adults by fusing with each other or injured myofibers, resulting in the formation of new myofibers [38]. To investigate the biological role of *Mat2a* in skeletal muscle, we utilized a C2C12 myoblast model to study its function, and the expression of MAT2A was knocked down by two siRNA (si*Mat2a*-1 and si*Mat2a*-2). Compared to control samples treated with non-targeting siRNA, the mRNA and protein levels of MAT2A were significantly reduced after *Mat2a* knockdown (Figure 2A,B). The levels of SAM, generated by MAT2A, also significantly decreased (Figure 2C). To investigate the role of MAT2A in myoblasts’ differentiation, C2C12 cells were induced to differentiate with medium supplemented with 2% horse serum and treated with si*Mat2a* (Figure 2D). C2C12 cells with *Mat2a* knockdown exhibited impaired differentiation into myotubes after four days of induction (Figure 2E). This result demonstrated that *MAT2A* plays a significant role in modulating myoblast differentiation.

PF9366, an inhibitor of MAT2A, binds to an allosteric site on MAT2A, enhancing substrate affinity and reducing enzyme turnover [31]. In accordance with its pharmacological activity, PF9366 decreased SAM levels in C2C12 cells (Figure 2F). The number of myotubes decreased after four days of differentiation in C2C12 cells treated with PF6366 (Figure 2G,H). The inhibition of MAT2A activity had an impact on myoblast differentiation that was comparable to the effects observed when its expression levels were reduced, indicating that this process depends on enzyme activity.

### 3.3. PF9366 Inhibited Skeletal Muscle Repair after BaCl_2_-Induced Injury in Mice

**Figure 3:**
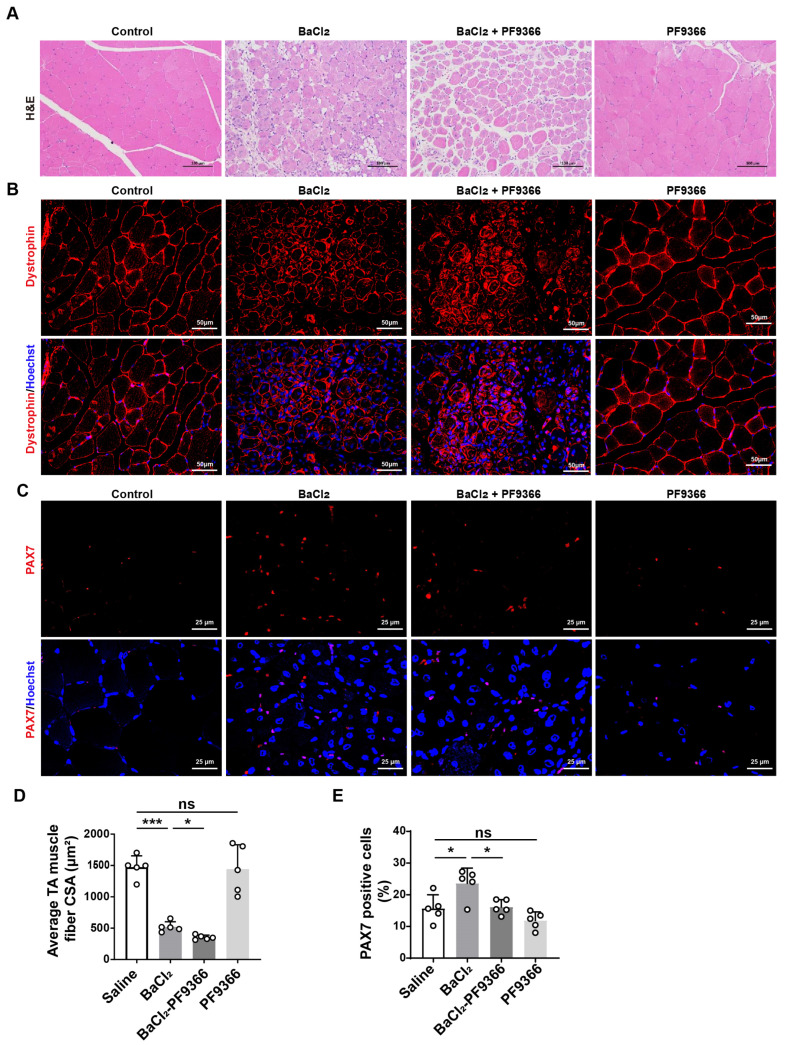
PF9366 inhibited skeletal muscle repair after BaCl_2_-induced injury *in vivo*. (**A**) H&E staining for tibialis anterior (TA) muscles of young mice (4 months, n = 4~5) treated with BaCl_2_, BaCl_2_ + PF9366, and PF9366 for 7 days. Saline was used as the control. Scale bar: 100 µm. (**B**) Immunofluorescence staining for dystrophin (red) and nuclei (blue) in TA muscles of young mice (4 months, n = 4~5) treated with BaCl_2_, BaCl_2_ + PF9366, and PF9366. Saline was used as the control. Scale bar: 50 µm. (**C**) Immunofluorescence staining for PAX7 (red) in TA muscles of young mice (4 months, n = 4~5) treated with BaCl_2_, BaCl_2_ + PF9366, and PF9366. Saline was used as the control. Scale bar: 25 µm. (**D**) Tibialis anterior (TA) cross-sectional area measured by immunofluorescence staining of dystrophin. (**E**) Quantification of the percentage of PAX7-positive cells in muscle of mice. Means were compared using two-way ANOVA. Data are presented as mean ± SD. * *p* < 0.05, *** *p* < 0.001.

The differentiation of myoblasts plays a crucial role in the regeneration process of skeletal muscle [39,40]. Considering the role of MAT2A in myoblast differentiation, we hypothesized that inhibition of MAT2A activity may decelerate skeletal muscle repair after injury. Intramuscular injection of BaCl_2_ induces skeletal muscle injury in mice, which will be repaired over time [41,42]. The treatment of the tibialis anterior (TA) muscle with BaCl_2_ resulted in necrosis of injured skeletal muscle fibers. Furthermore, it should be noted that the utilization of PF9366 exacerbated this injury. The administration of PF9366 alone did not impair skeletal muscle, but it exacerbated the BaCl_2_-induced skeletal muscle injury (Figure 3A). Immunofluorescence staining demonstrated that compared to BaCl_2_ alone, the combination of PF9366 and BaCl_2_ significantly reduced the mean cross-sectional area of muscle fibers by 32.2% (Figure 3B,D). PF9366 alone did not induce any skeletal muscle damage (Figure 3A,B,D). Considering the pivotal role of skeletal muscle satellite cells in skeletal muscle regeneration and their differentiation into myoblasts [43], we further investigated the dynamics of stem cell populations during the process of skeletal muscle regeneration. PAX7 is a marker for satellite cells that are involved in skeletal muscle regeneration [44]. PAX7-positive cells were observed in the skeletal muscle of young mice following a seven-day treatment with BaCl_2_ (Figure 3C,E). Remarkably, treatment with PF9366 resulted in a 43.4% reduction in PAX7-positive cells within the skeletal muscle, compared to control mice treated with BaCl_2_ (Figure 3C,E). We speculated that the activity of MAT2A may be suppressed by PF9366, thereby potentially impeding the differentiation process of stem cells and ultimately impacting skeletal muscle regeneration.

### 3.4. SAM Supplementation Promotes Skeletal Muscle Repair after BaCl_2_-Induced Injury in Mice

**Figure 4:**
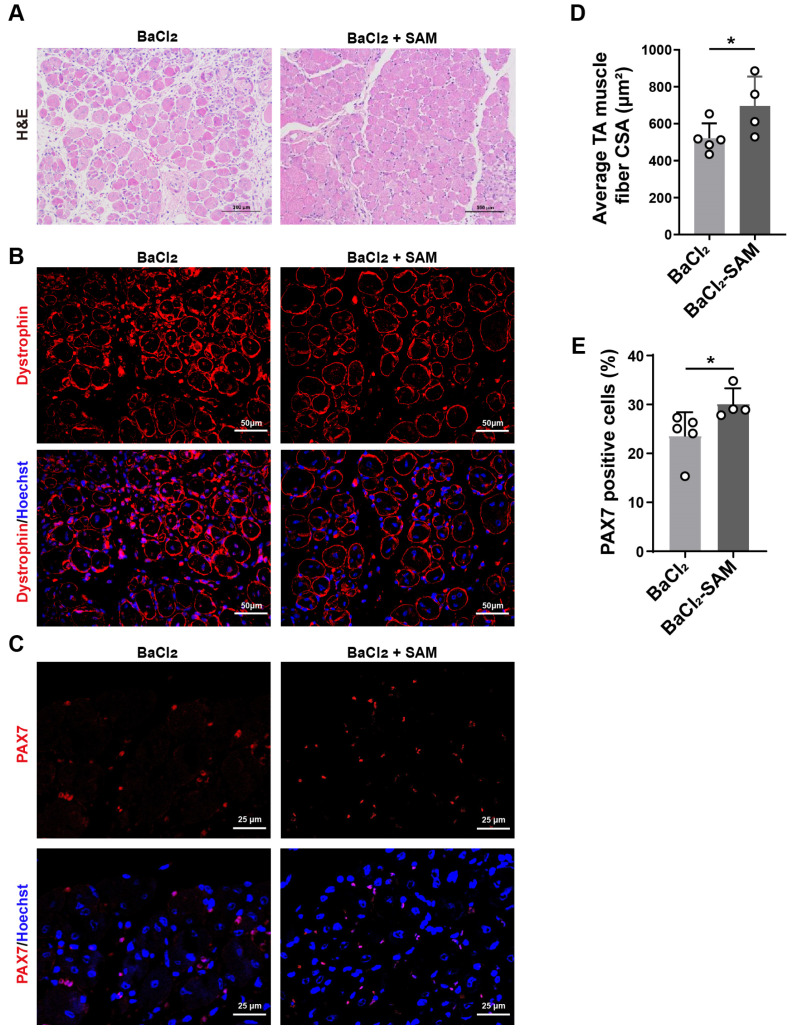
Supplementation of SAM promoted skeletal muscle repair after BaCl_2_-induced injury. (**A**) H&E staining for tibialis anterior (TA) muscles of young mice (4 months, n = 4~5) treated with BaCl_2_ and SAM for 7 days. Saline was used as the control. Scale bar: 100 µm. (**B**) Immunofluorescence staining for dystrophin (red) and nuclei (blue) in TA muscles of young mice (4 months, n = 4~5) treated with BaCl_2_ and SAM. Saline was used as the control. Scale bar: 50 µm. (**C**) Immunofluorescence staining for PAX7 (red) in TA muscles of young mice (4 months, n = 4~5) treated with BaCl_2_ and SAM. Saline was used as the control. Scale bar: 25 µm. (**D**) Tibialis anterior (TA) cross-sectional area measured by immunofluorescence staining of dystrophin. (**E**) Quantification of the percentage of PAX7-positive cells in muscle of mice. Means were compared using two-way ANOVA. Data are presented as mean ± SD. * *p* < 0.05.

The primary role of MAT2A is to facilitate the synthesis of SAM [22]. Therefore, we administered SAM to the mice via their drinking water. After SAM supplementation, we observed a decrease in skeletal muscle injury induced by BaCl_2_ (Figure 4A,B). Additionally, SAM supplementation resulted in a 25.1% increase in the mean cross-sectional area of muscle fibers compared to BaCl_2_ alone (Figure 4B,D). In addition, supplementation with SAM led to a 30.7% increase in the number of PAX7-positive cells (Figure 4C,E). These results suggest that SAM might facilitate the repair of BaCl_2_-induced skeletal muscle injury. 

### 3.5. Inhibition of MAT2A Activity Promotes Fas Expression

**Figure 5:**
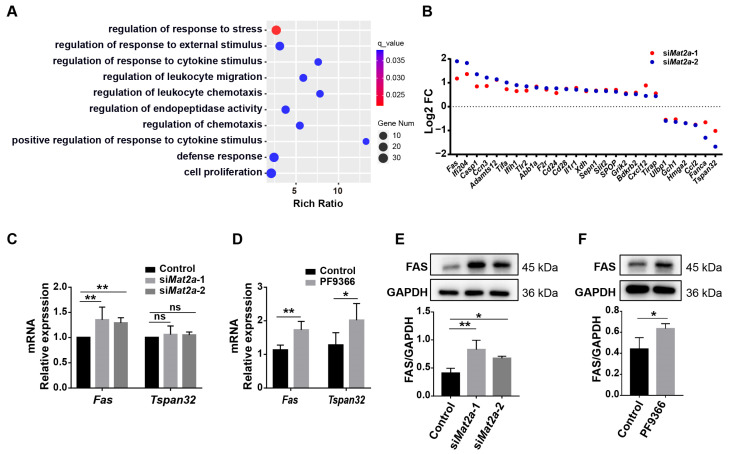
MAT2A inhibition increased the expression of Fas in C2C12 cells. (**A**) GO enrichment of DEGs in C2C12 cells treated with si*Mat2a*. (**B**) Fold changes of the expression of 27 DEGs regulating the response to stress. (**C**,**D**) qPCR was used to measure the mRNA levels of *Fas* and *Tspan32* in C2C12 cells treated with si*Mat2a* and PF9366. Comparison of means by two-way ANOVA. (**E**) Western blotting for the protein expression of Fas in C2C12 cells with *Mat2a* knockdown. Protein levels, normalized to GAPDH, were estimated by densitometry. Comparison of means by one-way ANOVA. (**F**) Western blotting for the protein expression of Fas in C2C12 cells treated with PF9366. Protein levels, normalized to GAPDH, were estimated by densitometry. Student’s *t*-test was used to compare means. Each assay was conducted in triplicate. Data are presented as mean ± SD. “ns” indicates not significant (*p* > 0.05), * *p* < 0.05, ** *p* < 0.01.

We next turned back to decipher the molecular mechanism by which MAT2A influenced the differentiation of myoblasts. Transcriptomic sequencing was conducted on C2C12 cells with *Mat2a* knockdown to elucidate how MAT2A regulates myoblast differentiation. The enrichment analysis of GO biological processes revealed a significant enrichment of DEGs in regulating the response to stress (Figure 5A). There were 27 genes regulating the response to stress. Of the 25 genes investigated, the expression of *Fas* expression was significantly upregulated and the expression of tetraspanin 32 (*Tspan32*) was significantly downregulated, exhibiting the greatest alterations in expression levels (Figure 5B). However, the results of qPCR revealed that neither knockdown *Mat2a* nor PF9366 treatment decreased *Tspan32* expression (Figure 5C,D), despite the predictions of the transcriptome analysis. Consistent with the results of the transcriptome analysis, MAT2A knockdown increased the mRNA and protein levels of Fas (Figure 5C,E). Moreover, inhibition of MAT2A activity enhanced the mRNA and protein levels of Fas (Figure 5D,F). Thus, we hypothesized that MAT2A might regulate myoblast proliferation and differentiation by modulating the expression of the Fas gene.

### 3.6. Inhibition of MAT2A Expression or Activity Suppressed Proliferation and Promoted Apoptosis of Myoblasts

**Figure 6:**
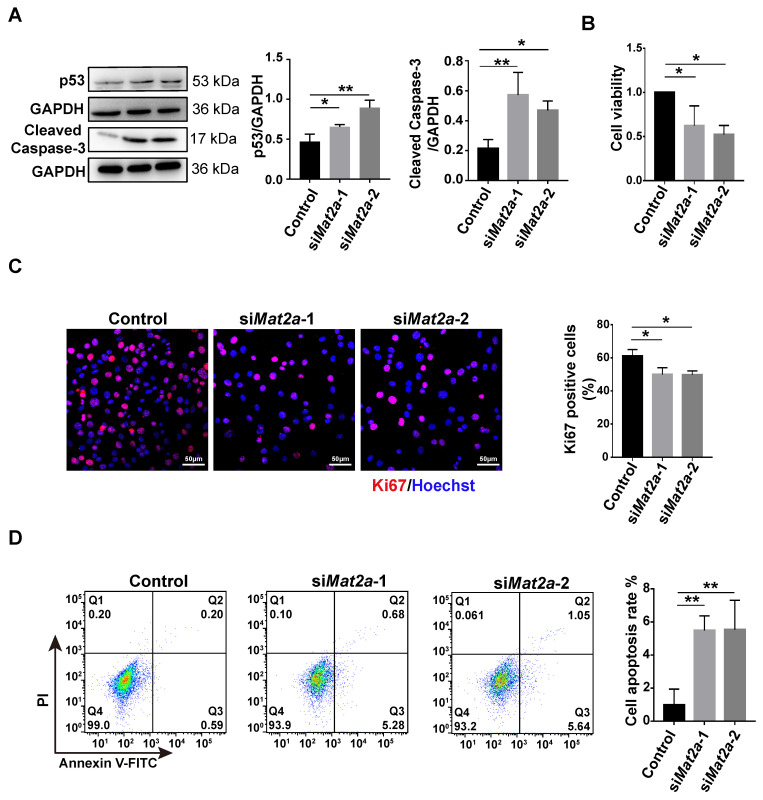
Knockdown of MAT2A inhibited the proliferation and induced apoptosis in C2C12 cells. (**A**) Western blotting for p53 and cleaved caspase-3 in C2C12 cells treated with si*Mat2a*. Protein levels, normalized to GAPDH, were estimated by densitometry. Means were compared using one-way ANOVA. (**B**) MTT for the viability of C2C12 cells treated with si*Mat2a* for 48 h. Statistical analysis by one-way ANOVA. (**C**) Immunofluorescence staining for Ki67 (red) and nuclei (blue) in C2C12 cells treated with si*Mat2a* for 48 h. Scale bar: 50 µm. One-way ANOVA was used to compare the percentage of Ki67-positive cells. (**D**) Annexin V-FITC/PI flow cytometric detection of apoptosis in C2C12 cells treated with si*Mat2a*. The percentage of apoptotic cells was compared using one-way ANOVA. Each assay was conducted in triplicate. Data are presented as mean ± SD. * *p* < 0.05, ** *p* < 0.01.

**Figure 7:**
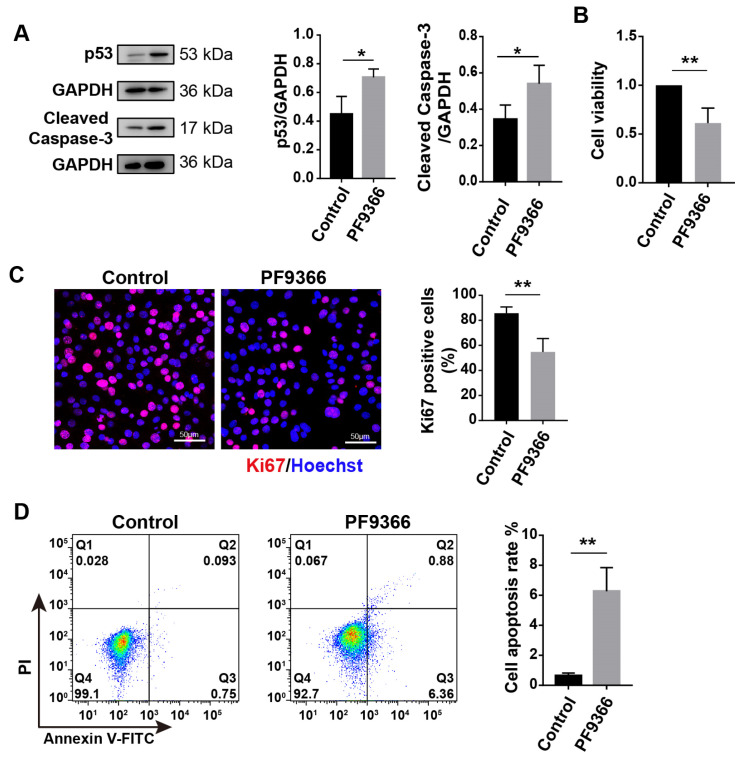
PF9366 inhibited the proliferation and induced apoptosis in C2C12 cells. (**A**) Western blotting for p53 and cleaved caspase-3 in C2C12 cells treated with PF9366. Protein levels, normalized to GAPDH, were estimated by densitometry. The Student’s *t*-test was used to compare means. (**B**) MTT for the viability of C2C12 cells treated with PF9366 for 48 h. The Student’s *t*-test was used to compare means. (**C**) Immunofluorescence staining for Ki67 (red) and nuclei (blue) in C2C12 cells treated with PF9366 for 48 h. Scale bar: 50 µm. The Student’s *t*-test was used to compare the percentage of Ki67-positive cells. (**D**) Annexin V-FITC/PI flow cytometric detection of apoptosis in C2C12 cells treated with PF9366. The Student’s *t*-test was used to compare means. Each assay was conducted in triplicate. Data are presented as mean ± SD. * *p* < 0.05, ** *p* < 0.01.

The transcription level of Fas is regulated by p53 [29,30]. The downregulation of MAT2A was found to result in an upregulation of p53 protein levels (Figure 6A). Therefore, we hypothesized that MAT2A deficiency or inhibition of MAT2A activity may induce myoblast apoptosis. We initially investigated the impact of MAT2A knockdown on cellular proliferation. The MTT assay revealed that C2C12 cells’ viability decreased by 37.7~47.7% after *Mat2a* knockdown (Figure 6B). Ki67 is a cell proliferation marker, which is present throughout the cell cycle [45]. C2C12 cells with *Mat2a* knockdown exhibited a 10% decrease in the number of Ki67-positive cells compared to the control group (Figure 6C). Importantly, flow cytometry analysis revealed that *Mat2a* knockdown increased the proportion of apoptotic C2C12 cells compared to control samples (Figure 6D). In addition, *Mat2a* knockdown increased the level of cleaved caspase-3, an apoptotic marker, in C2C12 cells (Figure 6A). Consistent with *Mat2a* knockdown results, treatment with PF9366 also increased the expression of p53 in C2C12 cells (Figure 7A). PF9366 decreased C2C12 cells’ viability (Figure 7B) and the number of Ki67-positive cells (Figure 7C). The proportion of apoptotic cells (Figure 7D) and the level of cleaved caspase-3 (Figure 7A) were enhanced in C2C12 cells treated with PF9366. These results suggest that inhibition of MAT2A activity suppresses the proliferation and induces the apoptosis of C2C12 cells, explaining the impaired cell differentiation in C2C12 cells with *Mat2a* deficiency.

## 4. Discussion

Skeletal muscle is the largest organ in the human body, responsible for maintaining posture, balance, and facilitating movement. Daily activities and exercise can cause minor muscle damage, and the metabolic activities of skeletal muscle maintain a dynamic balance of protein synthesis and degradation. Thus, the repair and regeneration capabilities of skeletal muscle are crucial for health. However, the repair capacity of skeletal muscle significantly declines with aging [46,47]. Identifying the genes or targets responsible for the decline in repair capabilities is crucial for developing interventions aimed at enhancing muscle repair in the elderly. Our multi-omics research identified the MAT2A gene as a critical factor in the diminished repair function in the elderly. Downregulation of MAT2A expression and a reduction in its catalytic activity both led to decreased muscle repair capabilities *in vivo*. Additionally, *in vitro* studies revealed that the decrease in MAT2A expression resulted in reduced proliferation and differentiation abilities of myoblasts and an increase in apoptosis. These findings illuminated the molecular mechanisms behind the impaired muscle repair capabilities in aging, offering important insights for potential therapeutic strategies.

In our study, we observed that knockdown of MAT2A impacted myoblast differentiation. This finding aligned with previous studies that have explored the role of satellite cells and myoblasts in the aging of skeletal muscle and the decline in its repair functionality [7]. Our *in vivo* study further validated the important role of MAT2A and its metabolic product SAM in skeletal muscle repair. Differentiation of myoblasts is a crucial process in the development and regeneration of skeletal muscles [48,49]. Hence, we investigated the role of MAT2A in skeletal muscle regeneration after injury. Skeletal muscle possesses a robust capacity for repair. In the BaCl_2_-induced mouse skeletal muscle injury model, a complete regeneration was observed after 28 days of injury [50]. Inhibition of MAT2A activity exacerbated BaCl_2_-induced skeletal muscle injury. The most severe injury was observed three days after induction, and the repair process started five days post-induction [51]. PF9366 treatment exacerbated BaCl_2_-induced skeletal muscle injury in mice, potentially by impairing skeletal muscle repair. BaCl_2_-induced skeletal muscle injury activated the repair mechanism, leading to myoblast differentiation into muscle fibers. We observed that inhibition of MAT2A activity resulted in a reduction in the population of skeletal muscle satellite cells involved in skeletal muscle regeneration following injury, whereas supplementation with SAM led to an augmentation in the number of skeletal muscle satellite cells. So, skeletal muscle regeneration requires a substantial amount of SAM after seven days of BaCl_2_ treatment. However, inhibition of MAT2A activity reduced SAM production in skeletal muscle and impaired the repair process. Therefore, more severe damage was noted in BaCl_2_-induced skeletal muscle injury after PF9366 treatment. Furthermore, our results demonstrated that SAM supplementation can facilitate the repair process after BaCl_2_-induced skeletal muscle injury. Inhibition of spermidine synthase restored intracellular SAM levels, improving muscle function and regeneration in elderly mice [20], which is consistent with our results. 

In our study of the mechanisms involved, we also discovered that knockdown of MAT2A led to increased apoptosis in myoblasts, consistent with earlier findings that satellite cells in elderly individuals are more prone to apoptosis [52]. The transcriptome sequencing analysis of C2C12 cells with *Mat2a* knockdown revealed a significant enrichment of DEGs that regulate the stress response, with a notable increase in Fas expression. Suppression of MAT2A expression or activity increased the expression of Fas. Fas, a member of the tumor necrosis factor (TNF) superfamily, is actively involved in apoptosis mediated by death receptors and is expressed in multiple tissues [28,53]. The results of our study validated that inhibition of MAT2A expression or activity can induce apoptosis and increase cleaved caspase-3 levels in C2C12 cells. The p53 regulates the expression of Fas [30]. We also observed that knockdown of MAT2A or inhibition of its activity were both found to enhance p53 expression in C2C12 cells. MAT2A, which produces SAM, is a crucial molecule essential for normal cell function and growth. Therefore, it can be speculated that *Mat2a* may regulate the proliferation and differentiation of myoblasts. These findings suggest that inhibition of *Mat2a* can trigger apoptosis via the p53–Fas signaling pathway, thereby impeding myoblast differentiation. 

Another unexpected result was that only 406 genes showed altered expression in both humans and mice, possibly due to various environmental factors. Mice in our study were reared in specific pathogen-free (SPF) environments, with age as the sole variable, while all other conditions were uniformly maintained. In contrast, the human dataset reflected not only age-associated variables but also lifestyle-associated factors, such as physical activity and dietary patterns, which impact skeletal muscle [54]. Consequently, we focused on a select group of differentially expressed genes (DEGs) that are more critical for skeletal muscle aging. Further analysis was performed on DEGs specifically related to cofactor metabolism. The heterogeneity of these differentially expressed genes across species underscores the significance of these shared genes for research. MAT2A is one such gene.

Our research identified MAT2A as a novel potential target for interventions aimed at enhancing skeletal muscle repair functions. The MAT enzyme family, consisting of subtypes MAT1A, MAT2A, and MAT2B, catalyzes the conversion of methionine and ATP to produce S-adenosylmethionine (SAM), the principal methyl donor [22]. Specifically, MAT2A is responsible for SAM synthesis in extrahepatic normal and cancerous tissues, whereas MAT1A is confined to liver and bile duct epithelial cells. Our bioinformatics analysis demonstrated a significant decline in MAT2A expression in the skeletal muscle of elderly humans and mice, supporting findings by Chen et al., who observed a gradual decrease in MAT2A expression in cultured vascular endothelial cells and the hearts of naturally aging mice [55]. These consistent findings strongly suggest that MAT2A plays a crucial role in aging and position it as a novel interventional target. Conversely, Rajabian et al. reported an increase in MAT2A expression in senescent myoblasts *in vitro* and used the lamin A knock-in (LAKI) mouse model to study MAT2A inhibition, and found protective roles of MAT2A inhibition on muscle regeneration in the LAKI mice model [56]. In contrast, our study found decreased MAT2A expression in aged muscle tissue from both mice and humans. This discrepancy could be attributed to the differences in the experimental models and contexts. The *in vitro* environment used by Rajabian et al. may not fully capture the complex regulatory mechanisms present *in vivo*. Additionally, the LAKI mouse model represents a specific pathological condition of premature aging, which might differ from the natural aging processes. These differences highlight the necessity of further investigation into the role of MAT2A across various models and contexts to fully understand its function in muscle aging and regeneration. The non-linear changes in MAT2A expression observed during aging, akin to the non-linear changes seen in serum albumin levels, emphasize the complexity of aging biology and the need for comprehensive mechanistic studies [57].

In summary, we found that MAT2A was conservatively downregulated in both humans and mice during skeletal muscle aging, and downregulation of MAT2A expression in aging skeletal muscle hindered muscle repair after injury. Inhibition of MAT2A activity regulated Fas expression, leading to myoblast apoptosis, and reduced myoblast numbers after skeletal muscle injury. Our study validated the pivotal role played by MAT2A in the process of skeletal muscle repair following injury. Our findings offer novel strategies to facilitate the repair process after skeletal muscle injury.

## Data Availability

The RNA-seq dataset in this study is available from the China National Center for Bioinformation (CNCB) with accession numbers of CRA012731 and CRA016255. Available online at: https://ngdc.cncb.ac.cn (accessed on 9 May 2024).

## Supplementary Materials

The following supporting information can be downloaded at: https://www.mdpi.com/article/10.3390/biom14091098/s1. Table S1: Gene annotation of mouse and human transcriptome data. Table S2: The expression of differentially expressed genes in muscle tissue of humans. Table S3: The expression of differentially expressed genes in muscle tissue of mice. Original images of Figure 2B, Figure 5E,F, Figure 6A and Figure 7A can be found in WB-original-images file.
