# Supplementary material for: Inhibition of MAT2A Impairs Skeletal Muscle Repair Function
Source: Biomolecules. 2024 Sep 2;14(9):1098. doi: 10.3390/biom14091098 (PMC11430595; doi:10.3390/biom14091098)
Supplement: Supplementary file 1 [file biomolecules-14-01098-s001.zip › Original Western-blot.pdf]

The Marker used in this study comes from Thermo Scientific™ (PageRuler™ Prestained Protein Ladder, 10 to 180 kDa, **Catalog number:** 26616)

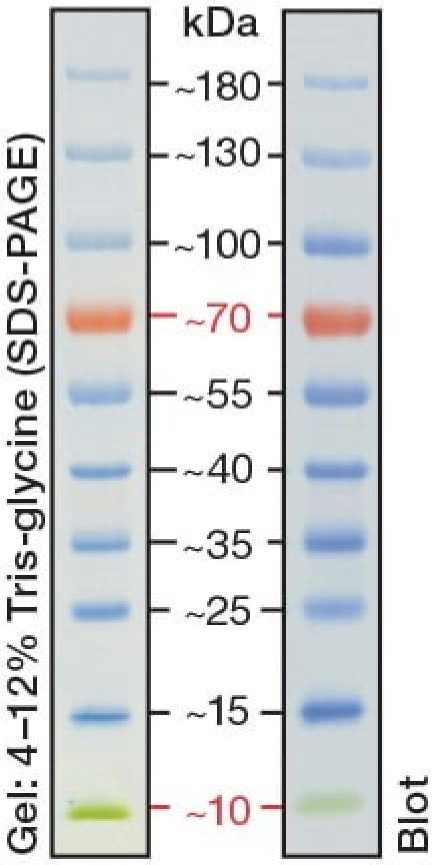

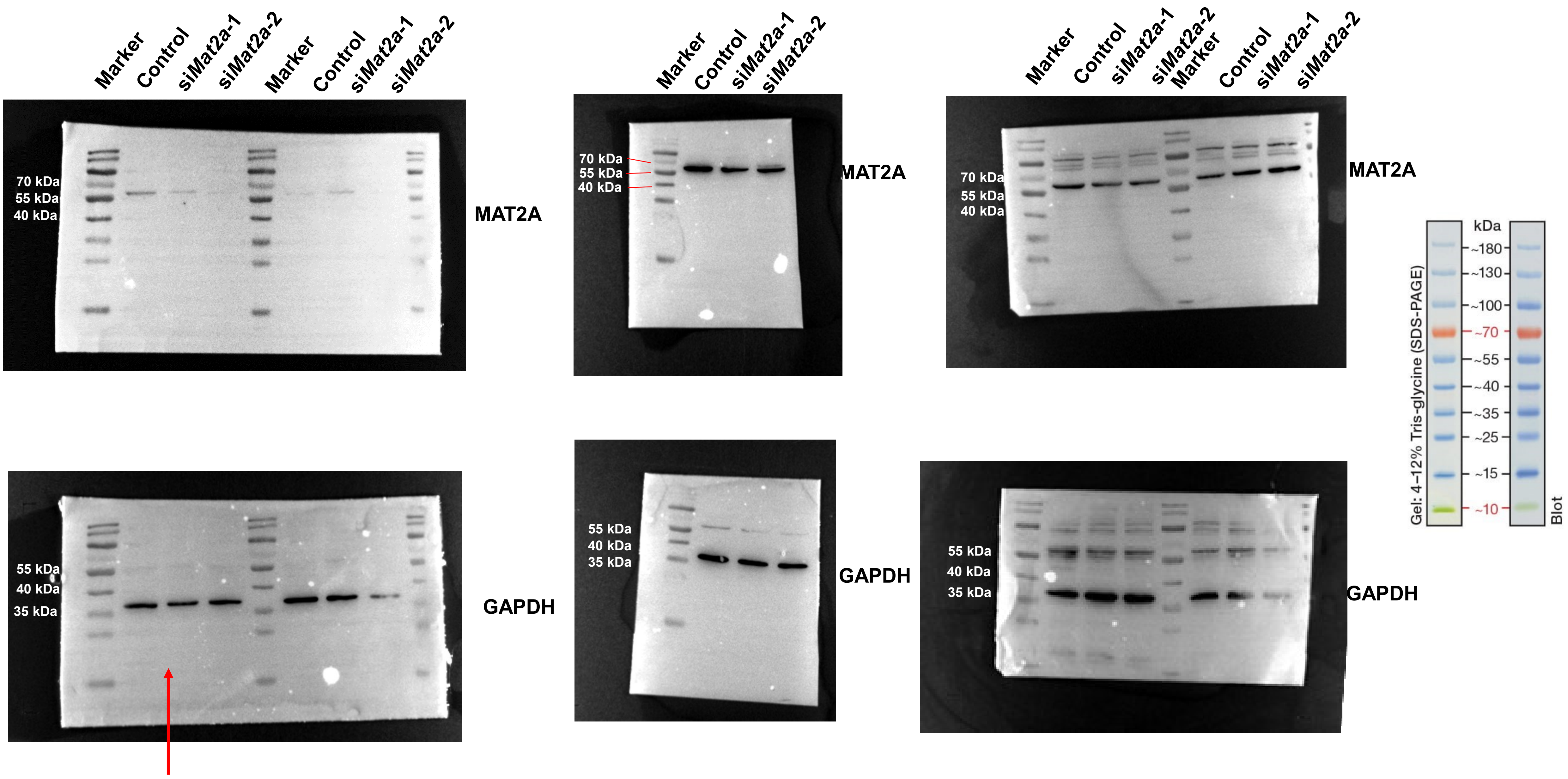

This original image corresponds to Figure 2B

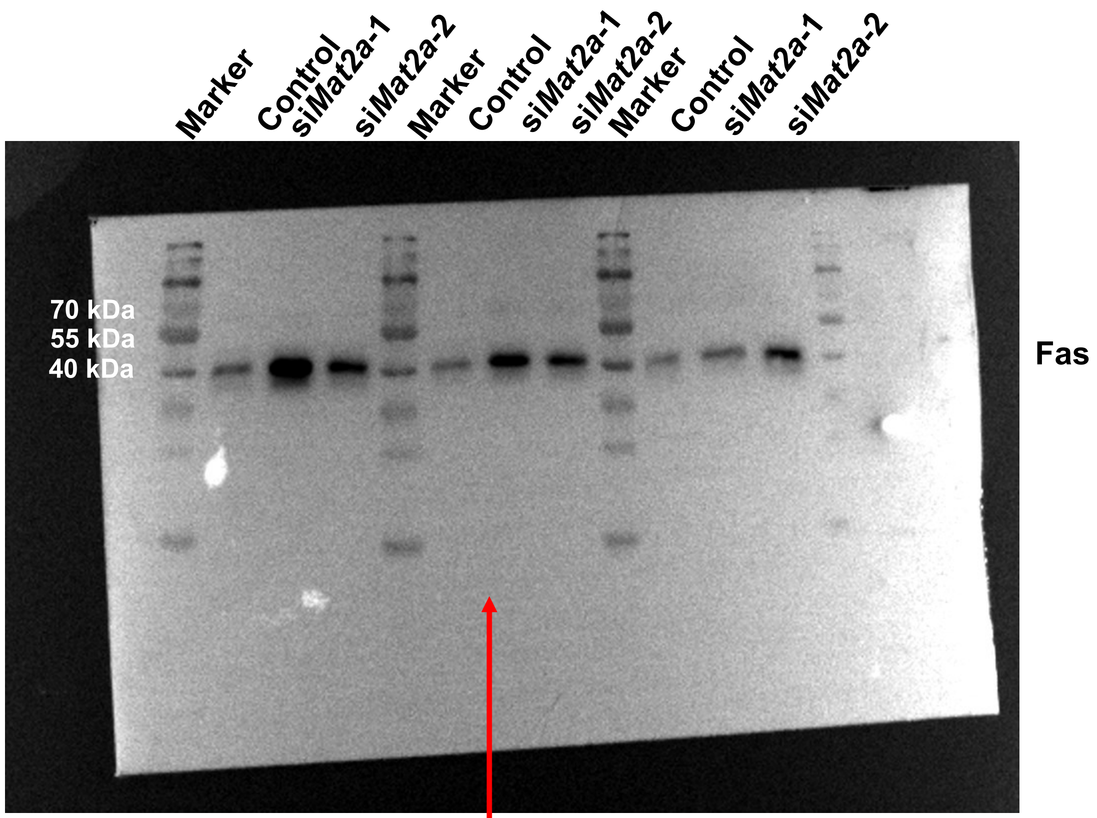

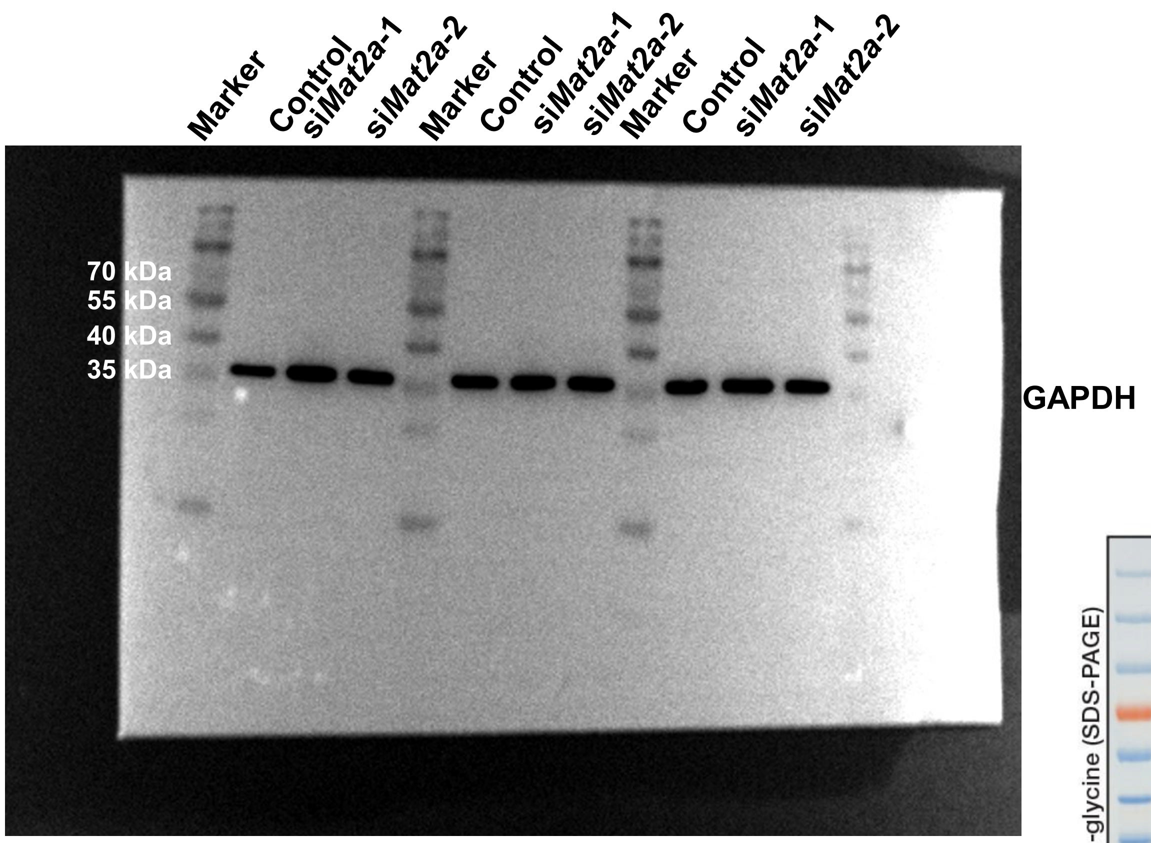

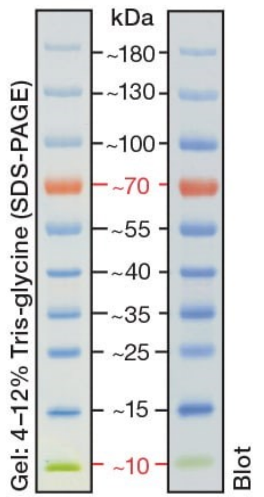

This original image corresponds to Figure 5E

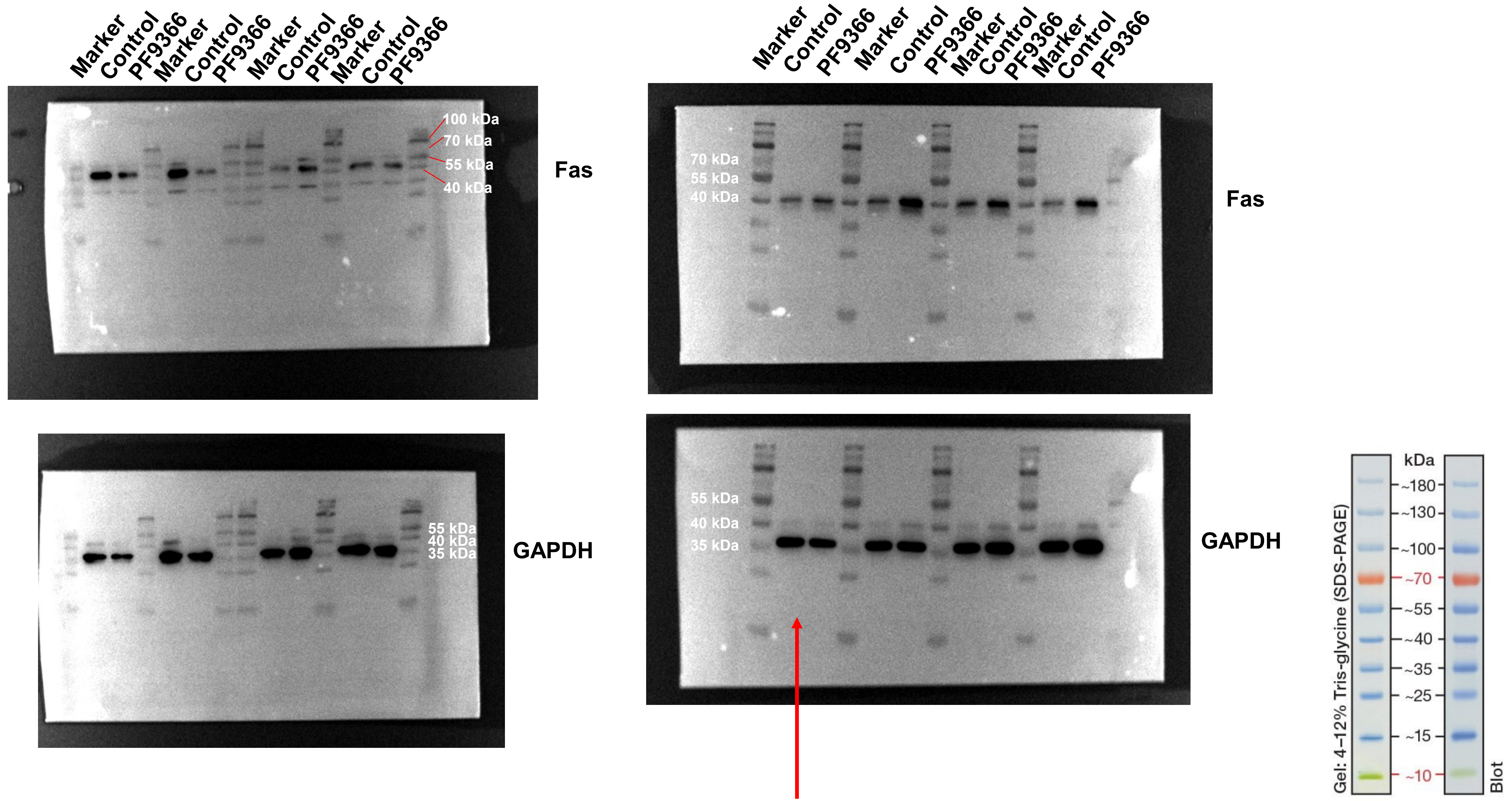

This original image corresponds to Figure 5F

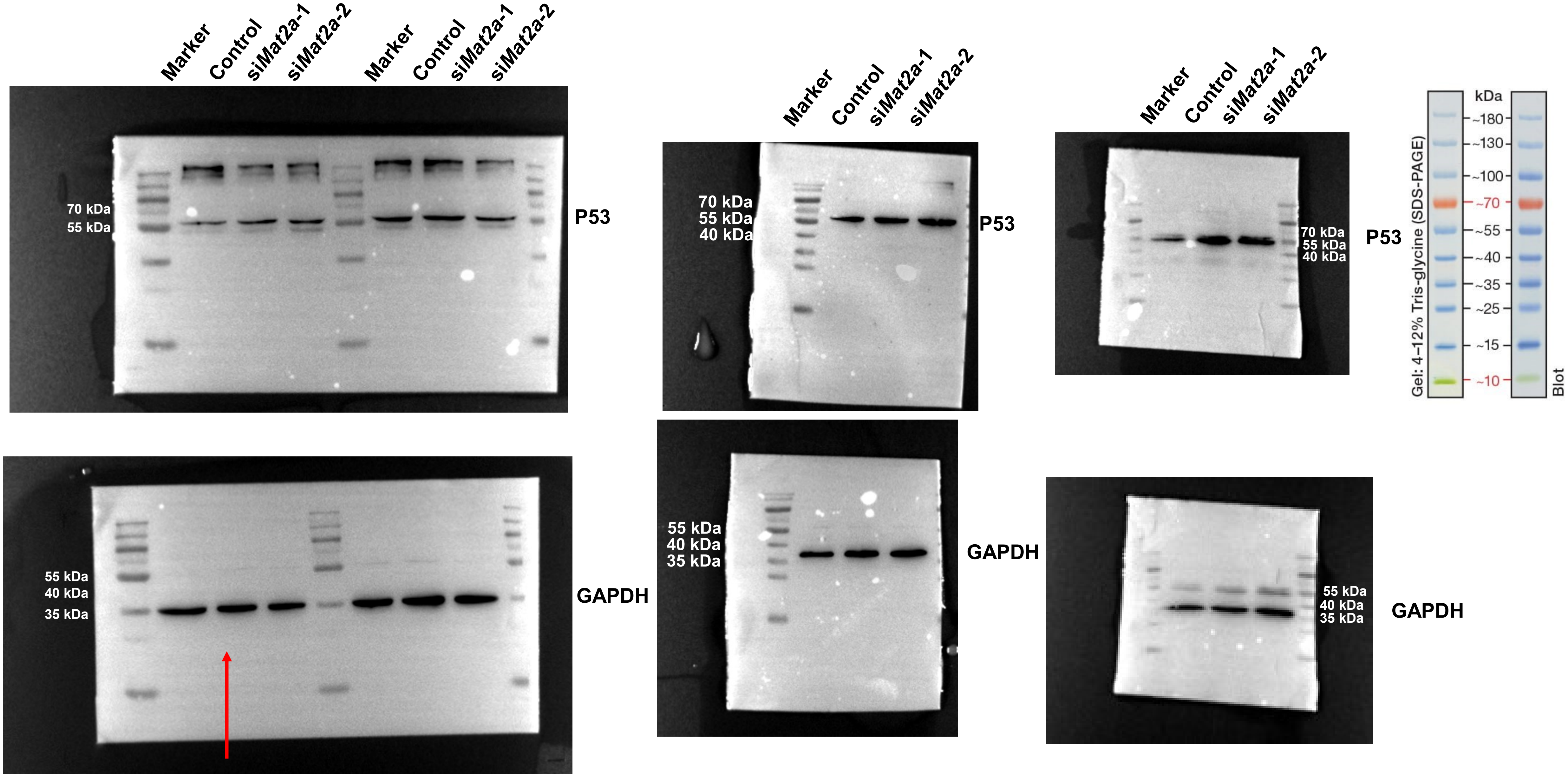

This original image corresponds to
Figure 6A P53

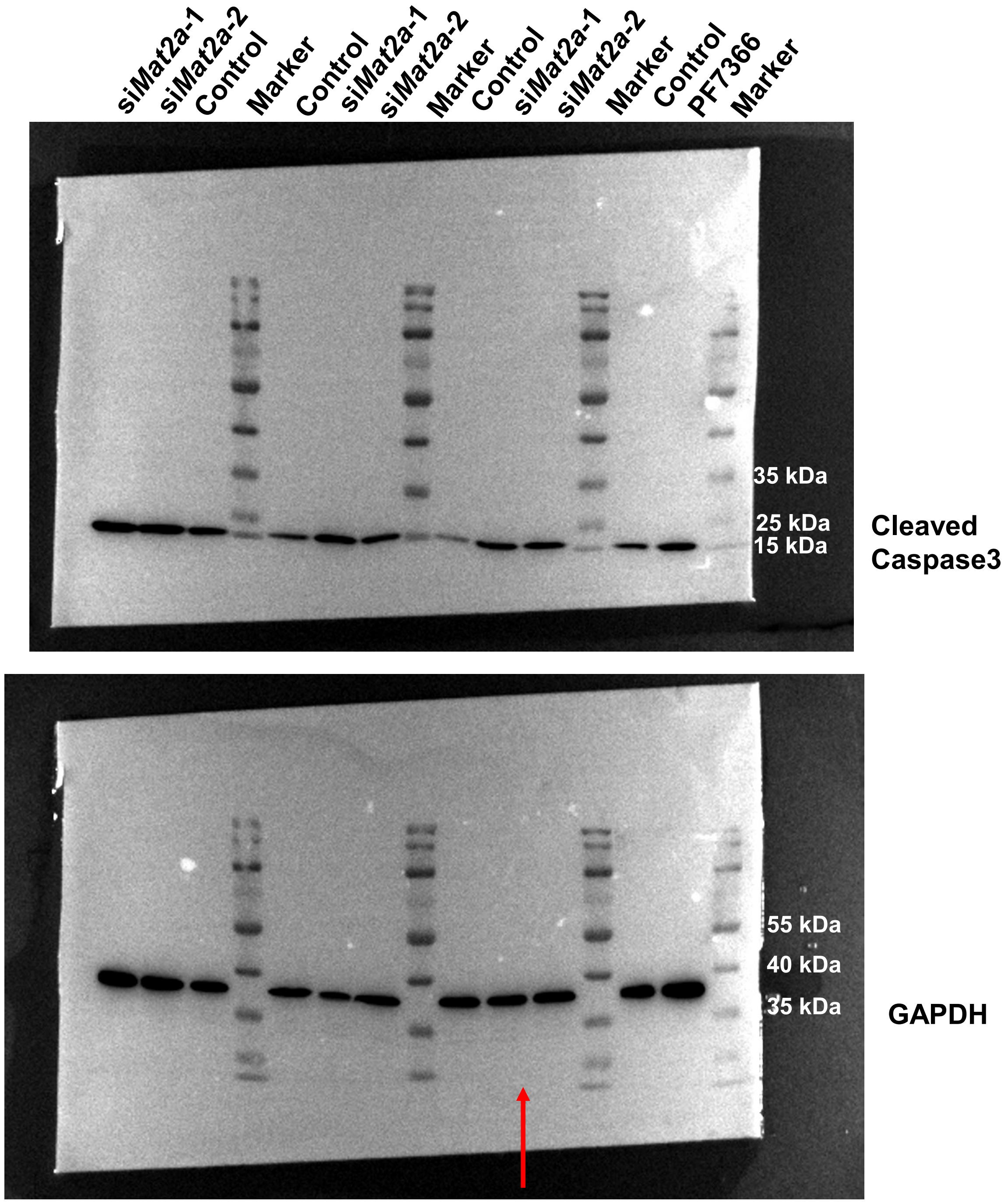

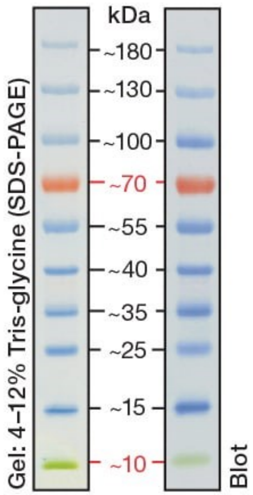

This original image corresponds to Figure 6A Cleaved Caspase3

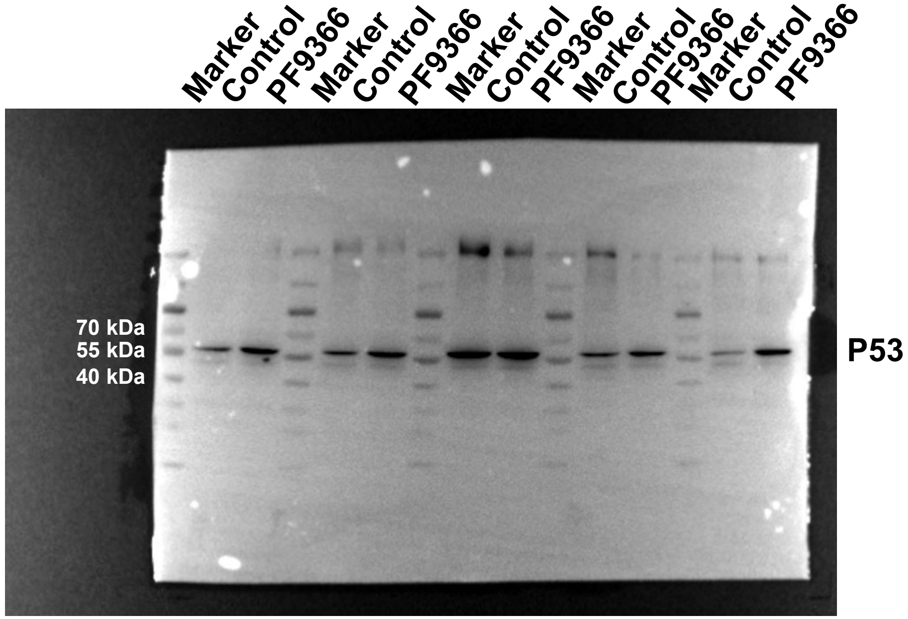

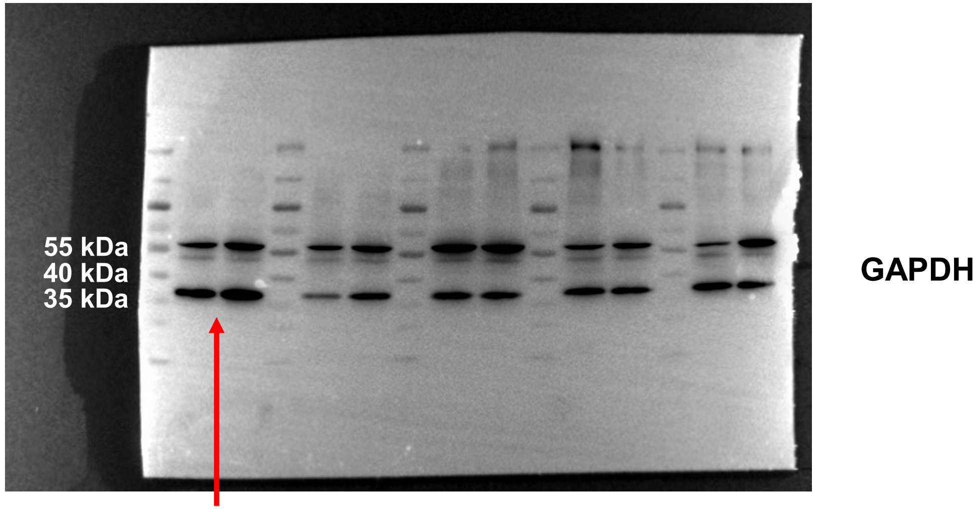

This original image corresponds to Figure 7A P53

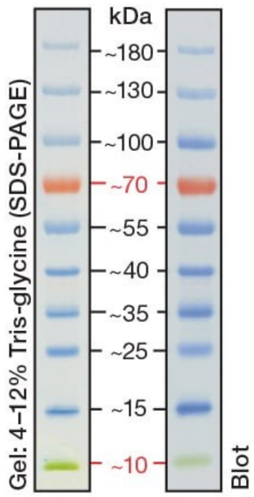

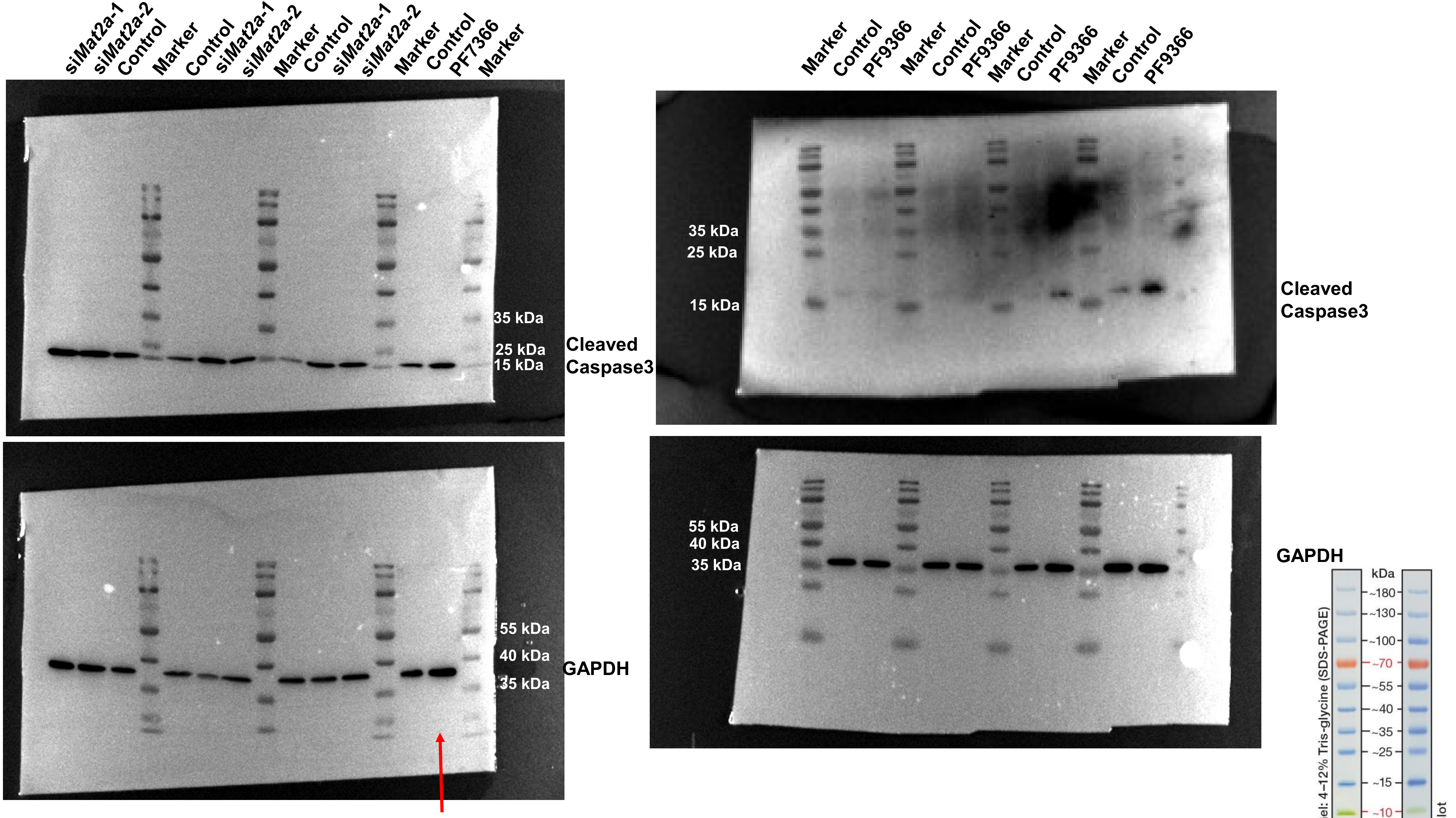

This original image corresponds to Figure 7A Cleaved Caspase3
